# How technical and situational cues affect impulse buying behavior in social commerce? Evidence from bored consumers

**DOI:** 10.3389/fpsyg.2024.1405189

**Published:** 2024-10-02

**Authors:** Yuhan Xue, Taiwen Feng, Chong Wu

**Affiliations:** ^1^School of Economics and Management, Harbin Institute of Technology, Harbin, China; ^2^School of Economics and Management, Harbin Institute of Technology, Weihai, China

**Keywords:** impulse buying, social commerce, boredom, S-O-R framework, fsQCA

## Abstract

**Introduction:**

With the rise of social media and web technologies, users are increasingly spending time on browsing and purchasing on social commerce, particularly during idle moments of casual scrolling. Social commerce applications with sophisticated social features and security measures may tend to attract a significant number of highly engaged users. The purpose of this study is to find out whether customers will be interested in the content posted on the applications and generate impulse consumption when they are bored.

**Methods:**

Drawing on stimulus-organism-response framework, this paper explores how technical cues and situational cues affect impulse buying behavior in social commerce applications and the mediating impact of consumer-perceived values. Data were gathered from 395 respondents who frequently utilize and have shopping experience on social commerce applications. The PLS-SEM and fsQCA were applied to formulate and test the proposed hypotheses.

**Results:**

The results of PLS-SEM reveal technical cues (ease of use, visual appeal and security) and situational cues (passing time and serendipity) positively influenced impulse buying. The results of fsQCA offer six solutions of different combinations of constructs which can lead to high impulse buying.

**Discussion:**

These findings may extend existing research on impulse buying behavior and consumer psychology, offering valuable insights for marketers. They also point towards strategies for more effectively encouraging impulse purchase in digital retail environments, particularly among consumers who are browsing out of boredom.

## 1 Introduction

Social commerce, an extension of e-commerce, has gained popularity alongside numerous advancements in new information technology. It integrates shopping functionalities into social media, enabling users to share experiences of products with each other. Examples of contemporary social commerce platforms include Xiaohongshu and WeChat. Social commerce not only allows consumers to browse and shop anytime and anywhere, but also provides an innovative sales channel for merchants. Shopping through social commerce has become highly influential in China. As of December 2023, the number of online shopping users reached 915 million, constituting 83.8% of all Chinese internet-users (China Internet Network Information Center, 2024).

While people use social media primarily to socialize and pass time rather than to shop, so their purchasing behavior often lacks clear goals and can be classified as “impulse buying” (IB) (Pookulangara and Shephard, 2013). Business activities on social media have been shown to help over 70% of manufacturers increase sales (Setyani et al., 2019), with more than 40% of online shopping driven by IB (Zafar et al., 2020). This growing focus on leveraging social media for online retail underscores the importance of IB as a key issue for marketers.

Previous research on IB has shown that retail environments can significantly impact consumer behavior. Hsin Chang and Wen Chen (2008) found that store characteristics, such as a relax and comfortable atmosphere, are conducive to IB. Chen and Yao (2018) demonstrated that website quality and designs can trigger IB by enticing consumers with visually appealing layouts. Additionally, “shopping values” are a pivotal determinant in the realm of IB. The primary drivers of online shopping, namely, utilitarian value and hedonic value, play a crucial roles in predicting consumption patterns (To et al., 2007). Utilitarian value, which is characterized by rational and practical considerations, involves consumers purchasing products for their practical utility. Conversely, hedonic value entails consumers purchasing products for emotional satisfaction and entertainment. Both values can affect individuals behavior in social commerce (Busalim et al., 2021).

IB sometimes is often considered a form of “emotional purchasing.” Research has indicated that positive emotions, such as pleasure, can increase IB (Parsad et al., 2021; Chen et al., 2022). However, few studies have examined the impact of other strong emotions, such as anxiety or boredom. The prevalence of remote work and reduced face-to-face interactions due to the impact of COVID-19 has heightened the likelihood of individuals experiencing boredom after completing daily tasks. Seeking stimulation and social connection, many turn to social media to escape feelings of boredom. These individuals are a worthy focus of research as potential consumers with significant amounts of time to browse social commerce platforms. Sundström et al. (2019) explored the connection between boredom and IB qualitatively. However, existing research lacks insight regarding how different actors combine to influence IB quantitatively. This study aims to fill that gap by investigating the relationship between boredom and IB using both qualitative and quantitative approaches.

Boredom is not limited to situations where social interaction is reduced due to exceptional circumstances such as global pandemics (Carsten et al., 2024). Indeed, in everyday life, people also experience moments of boredom, such as during commuting or in their leisure time at home. Many use their mobile devices to pass the time during these moments, where they may encounter a strong desire to make a purchase (Bozci, 2020). Though studies have consistently shown that people often make impulsive purchases on trusted online platforms (Nguyen-Viet and Nguyen, 2024), few have specifically examined consumer behavior as it relates to feelings of boredom. Hence, several research questions (RQs) remain:

RQ1: Do consumers engage in IB when they feel bored?

RQ2: What are the relationships among technical cues, situation cues and IB? How do shopping values influence IB among bored consumers?

To address these questions, we used partial least squares-structural equation modeling (PLS-SEM) and fuzzy-set qualitative comparative analysis (fsQCA) to explore the relationships among several influential factors. PLS-SEM reveals linear relationships among indicators from a quantitative perspective, while fsQCA reveals broad causal relationships among variables (Jiang et al., 2024). Combining these approaches allowed us to identify potential causes of high IB with different combinations of variables. Additionally, fsQCA can be used to test the validity of symmetric analyses. These methods facilitated an objective and accurate exploration of IB in the context of bored consumers browsing social commerce platforms. This study makes two main contributions. First, it introduces a research paradigm based on the stimulus-organism-response (S-O-R) framework to better explain IB in social commerce. Second, it expands consumer behavior theory by highlighting the role of boredom and identifying unique combinations of factors that drive high levels of IB.

The remainder of this paper is structured as follows. Section 2 reviews the theoretical background on social commerce applications, IB, boredom and the S-O-R framework. Section 3 outlines the hypothesis and research model of this study. Section 4 details the methodologies and data collection processes we used to support this work. Section 5 presents the results of the model and symmetric analyses. Section 6 discusses the theoretical and practical implications of our findings, the limitations and directions for further research. Section 7 provides concluding remarks.

## 2 Research background and theoretical foundation

### 2.1 Social commerce applications

Social commerce applications (SCAs) are virtual online communities where consumers gather to share, explore, and make purchases based on their individual preferences. These applications represent a refinement and enhancement of social commerce websites, emphasizing specialization and user-friendliness (Zhou et al., 2013). Acting as a bridge for consumer interactions, SCAs strategically blend online discussions with e-commerce functionality, incorporating message dialogs and embedded links that allow users to communicate with others about products and to make purchases instantly. SCAs also offer a highly personalized experience. Upon registration, users can select tags related to their preferences; big data algorithms then push tailored information based on these tags and the user’s browsing history. This unique push information ensures that users consistently interact with content that aligns with their interests.

SCAs also foster user engagement through features like sharing preferences, attaching tags, and providing purchase links within the app. Users can search for keywords, comment on posts, and interact with others, contributing to the establishment of extensive information-sharing networks with leader and follower relationships (Huang and Benyoucef, 2013). Additionally, SCAs offer remarkable accessibility – users can log in directly without the need for browser access (Yang et al., 2021). Their adaptability to mobile devices, particularly smartphones, eliminates the need for manual adjustments, providing users with a comfortable space for social and commerce activities on-the-go.

In essence, SCAs combine the advantages of social networking sites and e-commerce. SCAs prioritize user-to-user interaction (Olbrich and Holsing, 2011). They typically do not offer direct purchase channels rather rely on third-party links to facilitate actual shopping (Gonçalves Curty and Zhang, 2013). The selection of third-party links on SCAs is restricted, typically to well-known e-commerce platforms, ensuring a certain level of security for consumer accounts and payments.

SCAs are a product of e-commerce socialization but distinguishes itself in several key aspects. SCA users can create or join social groups aligned with their preferences, which fosters frequent interactions among users (Tajvidi et al., 2020). Throughout the shopping process, users actively support fellow consumers, offer insights into purchasing decisions, and aid in the selection of products that meet specific needs. In contrast, users of e-commerce platforms operate independently (Huang and Benyoucef, 2013). The entire process, from browsing shopping sites and making product comparisons to adding items to shopping carts and completing purchases, unfolds autonomously on SCAs. This direct, one-on-one interaction between buyers and sellers characterizes e-commerce, emphasizing the efficiency of transactions without the involvement of third parties.

### 2.2 Impulse buying

Impulse buying (IB) entails a sudden and powerful desire to make an unplanned purchase, often prompted by a stimulus (Cavazos-Arroyo and Máynez-Guaderrama, 2022). This stimulus may originate from products themselves (e.g., their functionality or usefulness) or external factors (e.g., the shopping atmosphere or visual appeal) (Akram et al., 2018). While scholars initially emphasized on offline IB in establishing this concept, the widespread popularity of social media and other internet tools has shifted focus toward understanding online IB (Han, 2023; Spiteri Cornish, 2020). Researchers have increasingly delved into the intrinsic factors of consumers as entry points to comprehend the mechanisms leading to IB in this context (Van Tran et al., 2023).

Stern (1962) classified IB into for types: pure, reminder, suggestive, and planned. This categorization continues to significantly influence research on IB (Rao and Ko, 2021). Pure IB occurs when consumers browse posts on SCAs aimlessly before deciding to purchase a certain product. Reminder IB takes place when consumers, while casually browsing SCAs, realize the need to replenish certain items soon (e.g., household consumable goods) and immediately purchase them. Suggestive IB occurs when consumers make purchases based on recommendations from other SCA users. Finally, planned IB occurs when a consumer seeks information on SCAs regarding a specific purpose for which they are shopping, ultimately deciding to make purchases based on promotions.

The rich array of text, images, and videos (or vlogs) on SCAs stimulates users both visually and psychologically, readily leading to impulsive purchases. Browsing on SCAs is often perceived as a continuous exploration activity, undertaken without specific goals, characterized by undirected and less-focused engagement. Users may turn to browsing on SCAs during periods of boredom, experiencing a form of “treasure hunting” that brings satisfaction as well as opportunities for IB. Previous studies have indicated that the mass active user base on SCAs facilitates frequent, convenient consumer interactions and information transfer, significantly impacting emotions and self-esteem, which makes users more prone to impulsive consumption (Thompson and Prendergast, 2015; Jin and Ryu, 2020). Psychologically, IB is spontaneous, pleasurable, and satisfying – it is thus emotion-driven and potentially irresistible (Setyani et al., 2019). Further, both positive and negative moods impact IB (Parsad et al., 2021; Pornpitakpan et al., 2017). Table 1 summaries the previous studies on factors influencing online IB.

**Table 1:** The previous study about IB.

Authors	Framework	Antecedents	Dependent variables	Research methods
Thompson and Prendergast (2015)	Five-factor model	Trait affect, Self-regulation	IB	Survey
Huang (2016)	S-O-R	Social capital, content attractiveness, peer communication, browsing activities, UBI	IB	Survey
Xiang et al. (2016)	S-O-R	Information fit-to-task, visual appeal, parasocial interaction	UBI	Survey
Pornpitakpan et al. (2017)		Service quality, mood	IB	Survey
Akram et al. (2018)		Website quality, promotion, credit card use	IB	Survey
Chen et al. (2019)	Signaling	Recommender related signals, product related signals, cognitive and affective trust, product affection	UBI	Survey
Sundström et al. (2019)		Boredom as shopping motivation	UBI	In-depth interviews
Setyani et al. (2019)	S-O-R	Personalization, shopping values	UBI	Survey
Jin and Ryu (2020)		Parasocial interaction, envy, social comparison	IB	Experiment
Spiteri Cornish (2020)		Regret, mental disengagement	IB	Experiment
Chen et al. (2021)		Information technology affordance, targets of identification	IB	semi-structured interviews and survey
Parsad et al. (2021)		Mood regulation, shopping values	IBT	Survey
Rao and Ko (2021)	S-O-R	Brand loyalty, shopping values	IB	Survey
Zafar et al. (2021)	Latent state–trait	Sentiment, observational learning, authenticity, IBT	UBI	Survey
Cavazos-Arroyo and Máynez-Guaderrama (2022)		IBT, normative evaluation, UBI	IB	Survey
Han (2023)		Brand, social reactions, checkout process	Trust, perceived risk, UBI	Survey
Van Tran et al. (2023)		Upward social comparison, benign, self-esteem	IB	Survey
This article	S-O-R	Technical and situational cues, shopping values	UBI	Survey and fsQCA

IB, Impulse buying; UBI, Urge to buy impulsively; IBT, Impulse buying tendency.

Determining actual IB in academic research is challenging due to the diverse perceptions individuals have regarding when consumption is impulsive (Luo, 2005). To address this challenge, researchers typically use the concept of “urge to buy impulsively” as a rational proxy to measure actual IB (e.g., Chen et al., 2021; Hewei, 2022; Wang et al., 2021). This urge represents “a state of desire experienced upon encountering an object in the environment” (Beatty and Elizabeth Ferrell, 1998, p. 172). Additionally, given that most transactions on a SCA platform are conducted through third-party external links, these transactions may be influenced by numerous uncontrollable factors. Investigating IB as a psychological urge can enhance the accuracy and rigorousness of research (Parboteeah et al., 2009). So, this viewpoint was adopted in the present study to define the point at which purchases are genuinely impulsive.

### 2.3 Boredom

Boredom, a state of low stimulation or unpleasant emotions that individuals experience independently (Mikulas and Vodanovich, 1993), is widely recognized as a prevalent social phenomenon. When individuals feel disinterested or disengaged, they often take action to alleviate this psychological state. Scholars suggest that boredom arises for two main reasons: either individuals have too much free time with no definable purpose for it, or they lack something appealing to occupy their attention at that moment (Kwon et al., 2020). People may turn to various distractions or engage in various behaviors to escape feelings of boredom.

SCAs combine social media and e-commerce functionalities to allow users to share their personal experiences and knowledge, continuously and often unintentionally recommending products to others. Boredom has been shown to increase consumers’ purchase intentions (Park et al., 2022), and SCAs are filled with enticing information that encourages consumption. With the widespread use of smartphones and social media, individuals are likely to rely on their phones when they feel bored. Consumers often browse content that interests them without noticing how much time has passed (Witowska et al., 2020). Their likelihood of engaging in IB increases with the amount of time they spend on SCAs.

Strong emotions can drive purchasing behavior, and decisions made during emotionally charged moments tend to be irrational (Lidholm et al., 2017). Emotion-driven consumption can be positive, as it can lift consumers’ moods, enhance their focus, and improve their life satisfaction. While previous studies have superficially explored the relationship between boredom and IB (Peng et al., 2022), there is little research specifically focusing on bored consumers browsing SCAs. This study aims to comprehensively investigate IB on SCAs among users who feel bored.

### 2.4 S-O-R framework

Previous scholars have explored whether boredom-related atmospheric cues can induce IB, however, further research is necessary due to the complexity of this relationship. This study applies the S-O-R paradigm, initially proposed by A. Mehrabian and Russell (1974) to explore the extent to which individual differences influence the connection between stimuli and responses in mental structures and processes. Within the S-O-R framework, stimuli (S) encompass a range of internal and external forces that provoke mental responses. In the context of management and commerce, common external factors include marketing factors (Liang et al., 2021), celebrity effects (Chen et al., 2021), and destination layouts (Loureiro et al., 2021). Common internal stimuli are cultural factors (Redine et al., 2022), personal traits (Setyani et al., 2019), and perceived intimacy (Li et al., 2022). These stimuli, whether acting singularly or collectively, directly impact the organism (O) – the carriers that induce fluctuations and alterations in personal emotions – ultimately influencing the final behavioral response (R).

The significant role of the S-O-R model in understanding consumer behavior and the retail is widely acknowledged among scholars. For example, Ma et al. (2020) employed the paradigm to elucidate the positive impact of both strong and weak social ties on consumers’ purchasing intentions. The S-O-R model also closely aligns with the psychological process of IB and has been extensively used in studies on both online and offline consumption. Drawing from the S-O-R framework, Ming et al. (2021) found that social presence positively affects trust and flow experience, leading to IB. Barros et al. (2019) shown that retail store ambience can positively affect consumers’ emotional responses within the S-O-R paradigm. This study leveraged the comprehensive applicability of this paradigm as a theoretical framework for investigating whether consumers engage in IB when experiencing boredom.

## 3 Hypothesis development and research model

### 3.1 Technical and situational cues

#### 3.1.1 Ease to use

SCAs, as very widely adopted consumer platforms, place a high value on ease of use (EU). EU is the degree to which people use a certain system (Davis, 1989), a fundamental technical cue influencing users’ engagement with SCAs. The user’s ability to seamlessly navigate and explore SCAs without hindrance incentivizes them to partake in social activities such as posting and purchasing. In simpler terms, a more user-friendly SCA lends a more efficient and enjoyable browsing experience, fostering increased user engagement. In a quantitative study, Lim and Ting (2014) found that EU positively impacts consumers’ purchase intentions in online group shopping settings. Nasution et al. (2019) reached the same conclusion in an empirical study.

When physical activities may not be feasible or preferable, consumers are inclined to turn to SCAs as a comfortable and cost-effective option to relieve boredom. When consumers engage with interfaces that are more user-friendly, they are more likely to be satisfied. Accordingly, a positive evaluation of usability positively influences IB. We hypothesize that:

*H1*: EU is positively associated with (a) perceived utilitarian value and (b) perceived hedonic value.

#### 3.1.2 Visual appeal

Visual appeal (VA) is a technical cue that users tend to prioritize when navigating SCAs. VA encompasses the presentation of information (e.g., colors, layout, imagery) on SCAs and gauges its effectiveness in capturing users’ attention (Parboteeah et al., 2009; Zheng et al., 2019). The information interface garners an initial impression from the user, significantly influencing the entire visual experience. In the online fashion industry in particular, high-quality visuals of products play a supervisory role, influencing consumers to preferentially invest in them (Chen et al., 2020). Browsing an esthetically pleasing interface evokes satisfaction in consumers that could lead to IB (Liu et al., 2013).

For SCA users, a high-quality visual presentation of text and image information serves a dual purpose – it not only captures their attention but also facilitates the swift identification of relevant details. This expedites the process of filtering out desired products, ultimately supporting IB (Chopdar and Balakrishnan, 2020). When bored consumers aim to pass time on SCAs, VA becomes particularly impactful. Visual stimulation requires minimal complex brain activities, allowing consumers to easily pass time while experiencing sensations of pleasure and accomplishment. It is in this process that consumers may encounter items of interest to purchase impulsively.

*H2*: VA is positively associated with (a) perceived utilitarian value and (b) perceived hedonic value.

#### 3.1.3 Security

Consumers assess the value of products on SCAs based on text, images, and posted comments. If SCAs lack robust technology safeguards for privacy protection, efficient delivery, and information services, consumers will lose trust and refrain from making purchases on the platform regardless of how effectively product-related information is conveyed. Security (SEC) on SCAs is thus a crucial technical cue. In the online context, SEC is described as the extent to which a website is protected from personal information leakage or hacking, encompassing concerns such as information theft and credit card fraud (Soltanpanah, 2012). For instance, Alipay, China’s largest online payment platform, guarantees full compensation in the event a personal account is compromised; this instills a certain level of confidence in its users.

Likewise, when SCAs ensure SEC, consumers worry less about the safety of their accounts and can focus more on consuming products. Consumers are more likely to trust SCAs when they sense more safety in the shopping process (Kim et al., 2008). In e-commerce research, scholars often assert that SEC is a primary obstacle preventing internet users from shopping online (Han and Kim, 2017; Kim, 2012; Lian and Yen, 2014). For example, Wu et al. (2012) determined that customers are concerned about suppliers’ abilities to protect or properly use their personal information both before and after transactions are made. Thus, SEC is a key factor influencing whether consumers engage in IB. A higher level of SEC on SCAs may correlate with increased consumer reliance on the platform. The following hypotheses were established accordingly.

*H3*: SEC is positively associated with (a) perceived utilitarian value and (b) perceived hedonic value.

#### 3.1.4 Passing time

Passing time (PT) refers to an individual’s inability to recognize changes in their external environment when they are immersed in a satisfying activity. When individuals browse SCAs in bouts of boredom, they can become engrossed in the experience, leading to a distortion of their sense of time passing (e.g., Bao and Yang, 2022; Hsu et al., 2012; Huang, 2016). When consumers feel bored, they tend to gravitate toward browsing posts on familiar applications. This engagement unfolds in two ways: first, big data algorithms push tailored content for user to peruse, and second, users interact with their peers. Both avenues serve as effective means for consumers to spend time in efforts to alleviate boredom. Experiencing PT, as a psychological state, while browsing SCAs may also encourage consumers to engage in IB (Wu et al., 2020). Therefore, this study proposes the following hypothesis:

*H4*: PT is positively associated with (a) perceived utilitarian value and (b) perceived hedonic value.

#### 3.1.5 Serendipity

Serendipity (SER), characterized by “lucky accidents,” refers to the unexpected discovery of something desirable or valuable by chance (Foster and Ford, 2003). SER does not occur in isolation; it depends on the environment in which individuals are located (Edward Foster and Ellis, 2014). Exploring SCAs may resemble a “treasure hunt,” offering the prospect of unexpected and captivating discoveries that can evoke waves of emotion and induce cognitive changes. In the realm of social commerce, SER occurs when consumers peruse products information and unexpectedly find something they desire. Big data systems strategically push content based on users’ preferences, making users are more likely to stumble upon something valuable or interesting when browsing posts without specific shopping goals, giving rise to experiences of SER (Park et al., 2007) and subsequent IB.

Encountering surprises in the shopping process also generates intrinsic enjoyment for consumers, creating a desire to continue seeking items of value (Grange et al., 2019). When consumers feel bored and begin to browse SCAs, SER may cause them to become more impulsive than usual. Therefore, this study proposes the following hypothesis:

*H5*: SER is positively associated with (a) perceived utilitarian value and (b) perceived hedonic value.

### 3.2 Mediation of perceived values

#### 3.2.1 Utilitarian value

Utilitarian value refers to the value consumers perceive in using SCAs to accomplish specific shopping tasks, reflecting a goal-oriented motivation (Arnold and Reynolds, 2003). Utilitarianism involves consumers’ objective and rational judgment of products, typically expressed in terms of function, usefulness, and price (Chen et al., 2019; Lee and Chen, 2021; Xu et al., 2020). It roughly gauges whether consumers’ use of SCAs enhance their purchasing efficiency. The technical and situational cues on SCAs help consumers explore and discover their preferences, thereby increasing the efficiency of product consumption. As a result, consumers may become more inclined to complete shopping tasks by browsing content on SCAs. Research has indicated that highly productive shopping sites enable consumers to browse a more diverse range of products, increasing the likelihood of identifying items of interest and prompting IB (Park et al., 2012). Quantitative research has further shown that utilitarian value positively influences purchase intentions or IB (To et al., 2007; Yang et al., 2021). Therefore, we hypothesize:

*H6*: utilitarian value is positively associated with IB.

#### 3.2.2 Hedonic value

Hedonic value (HV) refers to the pleasure and enjoyment consumers expect to gain from an activity like making a purchase, and is a key factor in shopping behavior (Ryan and Deci, 2000). It encompasses the satisfaction and happiness derived from shopping experience, and is influenced by elements like enjoyment, fantasy, and the pursuit of social status (Chen et al., 2022; Wang and Tsai, 2017; Xiang et al., 2016). When applied to SCAs, it signifies the satisfaction users derive from browsing. Gan and Wang (2015) suggest that utilizing social media to pass the time during moments of boredom can generate hedonic satisfaction through activities that evoke excitement (e.g., listening to music, watching videos). Prior studies emphasized that positive emotions have a crucial matter of IB (Chen et al., 2019; Verhagen and van Dolen, 2011; Wu et al., 2016). When consumers experience boredom, exposure to product-related content on SCAs can create a positive, pleasurable experience that may trigger IB. Therefore, this study proposes the following hypothesis:

*H7*: Hedonic value is positively associated with IB.

**Figure 1:**
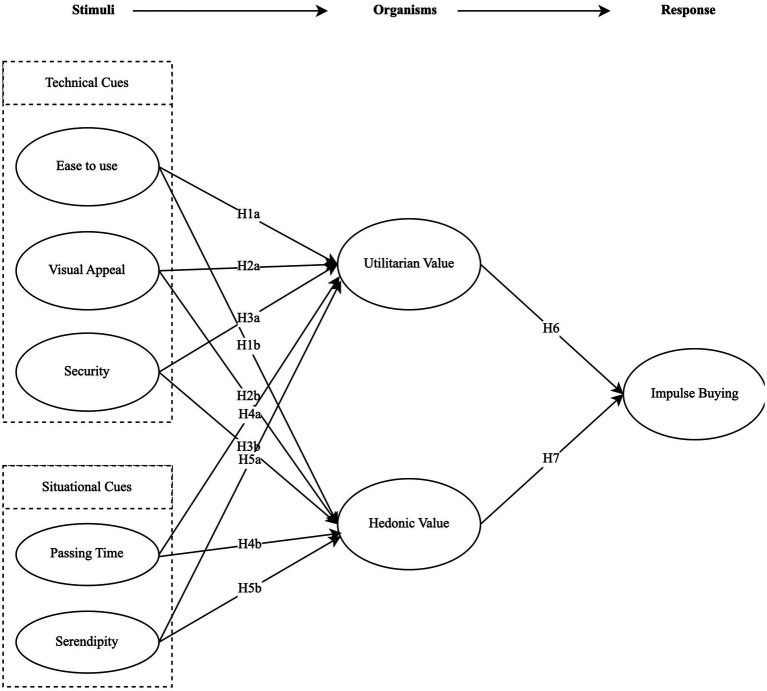
Research model.

The research model is shown in Figure 1.

## 4 Research method

### 4.1 Data collection

#### 4.1.1 Data collection method

This study utilized an online survey conducted on Wenjuanxing,^1^ China’s largest online questionnaire platform, to collect data. The target group consisted of a wide range of users experienced in using SCAs. Xiaohongshu, one of the most powerful social media platforms in China and a representative SCA, served as the primary research vehicle. The survey link was embedded within a content post and prominently featured on Xiaohongshu’s homepage, inviting users to participate. To encourage active engagement, respondents were offered a random-value red packet as an incentive for completing the questionnaire. Screening questions, including inquiries about leisure time spent browsing SCAs, were implemented; if respondents answered in the negative, the questionnaire was closed.

#### 4.1.2 Samples

A total of 433 users responded to the questionnaire. After removing invalid responses due to duplicate IP addresses or insufficient completion time, 395 valid questionnaires were retained. The gender distribution among respondents was relatively balanced and more than 95% were under the age of 40. The majority of participants had considerable experience with shopping online and using social media. Table 2 provides a summary of the respondents’ demographic information.

**Table 2:** Demographic profile.

Measure	Items	Frequency	Percentage (%)
Gender	Male	178	45.06
	Female	217	54.94
Age	Under 20	63	15.95
	20–25	128	32.41
	26–30	131	33.16
	31–40	51	12.91
	Over 40	22	5.57
Experience in social media use daily (hour)	Under 1	51	12.91
	1–2	88	22.28
	2–3	79	20.00
	3–4	124	31.39
	Over 4	53	13.42
Experience in online shopping (year)	Under 1	26	6.58
	1–3	103	26.08
	3–6	175	44.30
	6–10	60	15.19
	Over 10	31	7.85
Income (RMB/month)	Under 1,000	66	16.71
	1,001–2000	93	23.54
	2001–4,000	81	20.51
	4,001–8,000	110	27.85
	Over 8,000	45	11.39

### 4.2 Measures

All constructs were measured on multiple-item scales that had been adapted by previous researchers and modified to fit the context of this study. All items were scored on a seven-point Likert scale from “1 = strongly disagree” to “7 = strongly agree.” The scales were originally developed in English. The respondents’ native language is Chinese, so the translation-back-translation approach was used to ensure the consistency of meanings expressed in Chinese and English (Xi et al., 2023).

#### 4.2.1 Ease of use

This study utilized the EU scale developed by Shen (2012), which includes four items that to measuring the perceived convenience of SCAs (e.g., “Learning to use Xiaohongshu is easy.”) The Cronbach’s alpha for the scale was 0.847.

#### 4.2.2 Visual appeal

A three-item VA scale developed by Loiacono et al. (2007) was used to assess the degree of page attractiveness (e.g., “Xiaohongshu is visually pleasuring design.”). The Cronbach’s alpha for the scale was 0.788.

#### 4.2.3 Security

This study used the four-item scale to measure the degree of information protection developed by Gao et al. (2015) (e.g., “Xiaohongshu is truthworth.”). The Cronbach’s alpha for the scale was 0.834.

#### 4.2.4 Passing time

This study employed the four-item scale developed by Kim et al. (2017) to help users report their passing time experiences. The questionnaires include “I am easily attracted on Xiaohongshu.” The Cronbach’s alpha for this scale was 0.823.

#### 4.2.5 Serendipity

This study used three-item scale developed by McCay-Peet and Toms (2011) to measure of serendipity perceived by users when browsing (e.g., “I obtained unexpected insights on Xiaohongshu.”). The Cronbach’s alpha for the scale was 0.753.

#### 4.2.6 Utilitarian value

This study utilized the utilitarian value scale developed by Wu and Wang (2005), which is widely used to explore the usefulness of products to users. An example item is “Using Xiaohongshu can improve my performance.” The Cronbach’s alpha for the scale was 0.851.

#### 4.2.7 Hedonic value

This study used the hedonic value scale developed by Shen (2012), which commonly employed to measure user’s enjoyment of online resources (e.g., “I had fun using Xiaohongshu”). The Cronbach’s alpha for the scale was 0.723.

#### 4.2.8 Impulse buying

A four-item version of the IB scale designed by Parboteeah et al.’s (2009) is used to measure users’ instances of IB in the social commerce context. An example item is “I had the urge to buy products.” The Cronbach’s alpha for the scale was 0.826.

The summarized definition of these constructs is provided in Table 3. The measuring items for research model are listed in Appendix A.

**Table 3:** The summarized definition of constructs.

Construct	Definition	References
EU	The degree of convenience	Shen (2012)
VA	The degree of page attraction	Loiacono et al. (2007)
SEC	The degree of information protection	Gao et al. (2015)
FE	The degree of time distortion of users	Kim et al. (2017)
SER	The degree of surprise to users	McCay-Peet and Toms (2011)
UV	The degree of usefulness to users	Wu and Wang (2005)
HV	The degree of enjoyment to users	Shen (2012)
IB	The degree of whether they will impulse buying	Parboteeah et al.’s (2009)

EU, Ease of use; VA, Visual appeal; SEC, Security; FE, Flow experience; SER, Serendipity; UV=Utilitarian value; HV, Hedonic value; IB, Impulse buying.

### 4.3 Control variables

The absence of randomly assigned respondents in online questionnaires may increase the risk of common method bias, potentially impacting the results. This study incorporated control variables to measure SCA user characteristics, including gender, age, and income. Additional variables such as online shopping experience and daily time spent browsing SCAs were also included. It is important to note that control variables have no significant impact on other measured variables.

### 4.4 Data analysis techniques

#### 4.4.1 PLS-SEM

Smart PLS 3.0 was employed to operate the research model. Partial least squares (PLS) can function with non-normally distributed data comparing to covariance-based structural equation modeling (SEM) tools. Given its emphasis on prediction and theory development, which aligns with the research objectives, PLS was selected as the preferred approach. The research model was evaluated in a two-step analytical procedure: the measurement model and structural model (Hair et al., 2011).

#### 4.4.2 Fuzzy-set qualitative comparative analysis

In addition to the PLS-SEM approach, fsQCA was employed as a supplement to further enrich our theoretical examination. FsQCA is particularly well-suited for capturing complex, real-world scenarios and is considered more reliable and persuasive in empirical studies. Because it is not feasible for the measurement of mediating variables, seven independent variables (EU, VA, SEC, SER, PT, and utilitarian and hedonic values) were used to explore their relationships to IB as an outcome variable.

## 5 Results

### 5.1 PLS-SEM

#### 5.1.1 Measurement model

The measurement model was assessed to ensure the appropriate use of constructs, focusing on reliability and validity. Reliability was assessed by evaluating factor loadings. All constructs were reflective and exhibited factor loadings above 0.7, indicating good reliability. Convergent validity and discriminant validity were also assessed. Convergent validity, as determined by Cronbach’s alpha, composite reliability, and average variance extracted (Hair et al., 1998) were favorable results (Table 3) affirming strong convergent validity (Fornell and Larcker, 1981). Discriminant validity, which confirms the absence of correlations between constructs, was evaluated by comparing the square root of AVE with correlations between each construct and every other construct. As shown in Table 4, the square roots of AVE for each construct surpassed the correlations between other constructs, representing satisfactory discriminant validity.

**Table 4:** Reliability and convergent validity.

Constructs		Mean	SD	Factor loading	Cronbach’s alpha	CR	AVE
EU	EU1	5.534	1.086	0.821	0.847	0.895	0.682
	EU2	5.471	1.126	0.835			
	EU3	5.463	1.112	0.776			
	EU4	5.433	1.144	0.867			
VA	VA1	5.511	1.094	0.785	0.788	0.872	0.696
	VA2	5.542	1.091	0.829			
	VA3	5.524	1.080	0.885			
SEC	SEC1	5.534	1.103	0.842	0.834	0.889	0.667
	SEC2	5.441	1.057	0.806			
	SEC3	5.446	1.045	0.787			
	SEC4	5.534	1.049	0.830			
FE	FE1	5.542	1.075	0.839	0.823	0.883	0.653
	FE2	5.567	1.092	0.791			
	FE3	5.519	1.061	0.807			
	FE4	5.506	1.066	0.794			
SER	SER1	5.534	1.096	0.828	0.753	0.855	0.663
	SER2	5.441	1.095	0.851			
	SER3	5.446	1.082	0.822			
UV	UV1	5.435	1.124	0.811	0.851	0.9	0.691
	UV2	5.463	1.125	0.824			
	UV3	5.481	1.119	0.839			
	UV4	5.370	1.167	0.851			
HV	HV1	5.410	1.102	0.889	0.723	0.878	0.783
	HV2	5.453	1.065	0.881			
IB	IB1	5.438	1.047	0.771	0.826	0.881	0.65
	IB2	5.435	1.080	0.875			
	IB3	5.403	1.057	0.757			
	IB4	5.542	1.051	0.819			

EU, Ease of use; VA, Visual appeal; SEC, Security; FE, Flow experience; SER, Serendipity; UV, Utilitarian value; HV, Hedonic value; IB, Impulse buying.

As each response was provided by one person on a single questionnaire, there was a potential threat of common method bias. A method described by Liang et al. (2007) was used to assess the presence of this bias, according to the ratio of the average square of all substantive factor loadings to all method factor loadings. The results indicated that common method bias was not a major concern in this study.

#### 5.1.2 Structural model

**Figure 2:**
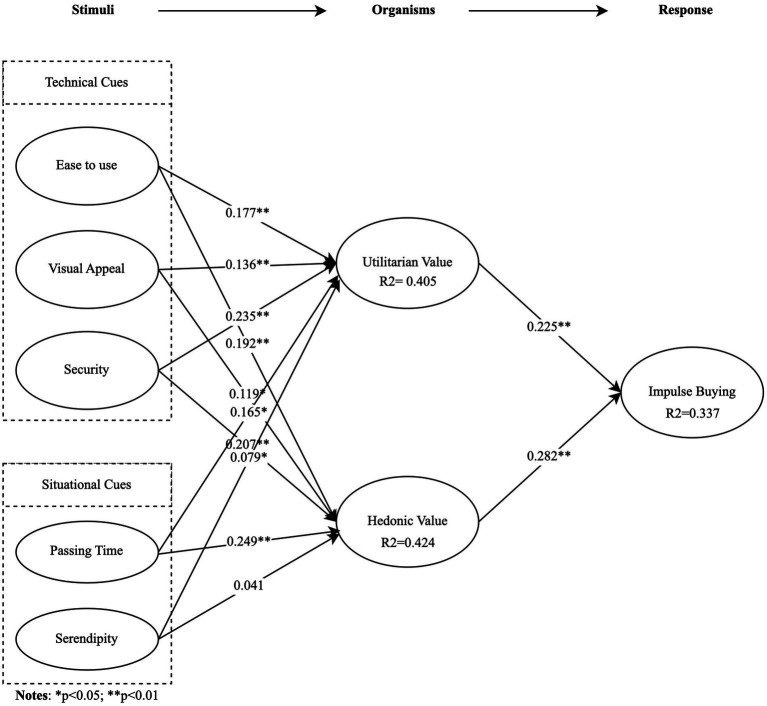
The result of research model.

Following the examination of reliability and validity, the hypotheses were tested using Smart PLS 3.0. As illustrated in Figure 2, the structural model results, including path coefficients and their significance, demonstrated significant relationships between each construct. These results largely support the hypotheses summarized in Table 5. All technical cues and situational cues significantly influenced a respondent’s utilitarian value, confirming H1a-H5a. Additionally, EU, VA, SEC, and PT significantly influenced a respondent’s hedonic value, supporting H1b-H4b. The weak relationship between SER and HV did not provide support for H5b. Finally, both utilitarian and hedonic values were found to significantly impact IB, supporting H6 and H7.

**Table 5:** Discriminant validity.

	EU	VA	SEC	FE	SER	UV	HV	IB
EU	**0.826**							
VA	0.172	**0.834**						
SEC	0.214	0.195	**0.817**					
FE	0.175	0.246	0.213	**0.808**				
SER	0.190	0.213	0.807	0.240	**0.814**			
UV	0.153	0.158	0.179	0.192	0.161	**0.831**		
HV	0.146	0.130	0.148	0.242	0.157	0.772	**0.885**	
IB	0.165	0.146	0.166	0.188	0.101	0.098	0.068	**0.807**

EU, Ease of use; VA, Visual appeal; SEC, Security; FE, Flow experience; SER, Serendipity; UV, Utilitarian value; HV, Hedonic value; IB, Impulse buying. bold values = the square root of relevant AVE.

#### 5.1.3 Mediation effects

Mediation effects were also examined. According to Hair et al. (2014), two requirements must be met for testing mediating variables. First, the independent variable should have a significant relationship with the mediating variable. Second, the mediating variable should have a significant relationship with the dependent variable. Variance accounted for values were calculated to determine whether the mediating variable constituted a full or partial mediating effect. As shown in Table 6, utilitarian value had a partial mediation between EU, VA, SEC, PT and IB, but has a full mediation between SER and IB. In addition, hedonic value exhibited partial mediation between the four independent variables and IB, with no effect between SER and IB.

**Table 6:** Hypotheses and results.

Hypotheses conclusions	Results
H1a: EU is positively associated with perceived UV.	Supported
H1b: EU is positively associated with perceived HV.	Supported
H2a: VA is positively associated with perceived UV.	Supported
H2b: VA is positively associated with perceived HV.	Supported
H3a: SEC is positively associated with perceived UV.	Supported
H3b: SEC is positively associated with perceived HV.	Supported
H4a: PT is positively associated with perceived UV.	Supported
H4b: PT is positively associated with perceived HV.	Supported
H5a: SER is positively associated with perceived UV.	Supported
H5b: SER is positively associated with perceived HV.	Not supported
H6: UV is positively associated with IB.	Supported
H7: HV is positively associated with IB.	Supported

EU, Ease of use; VA, Visual appeal; SEC, Security; FE, Flow experience; SER, Serendipity; UV, Utilitarian value; HV, Hedonic value; IB, Impulse buying.

### 5.2 Fuzzy-set qualitative comparative

#### 5.2.1 Selection and calibration of variable

The first step involved fuzzing the collected data within a value range of 0–1 and calibrating each variable using the mean of the scale data. Next, three qualitative thresholds were established corresponding to full membership, intermediate membership, and non-full membership at 95, 50, and 5% of the data frequency, respectively (Feng and Sheng, 2023; Zhang et al., 2024).

#### 5.2.2 FsQCA results

FsQCA includes two steps: necessary conditions and truth table algorithm operations. Following data calibration, necessary conditions analysis was conducted to determine whether an element served as a necessary casual condition for high/low IB levels. As shown in Table 7, none of the factors’ consistency values surpassed 0.9, indicating the absence of necessary conditions for high/low IB levels. Consistency values ranging from 0.59–0.66 implied that these factors cannot be deemed necessary for high/low IB levels.

**Table 7:** Mediation test.

Constructs	VAF	Effect
EU → UV → IB	0.30	Partial mediation
EU → HV → IB	0.37	Partial mediation
VA → UV → IB	0.32	Partial mediation
VA → HV → IB	0.34	Partial mediation
SEC → UV → IB	0.32	Partial mediation
SEC → HV → IB	0.34	Partial mediation
PT → UV → IB	0.26	Partial mediation
PT → HV → IB	0.40	Partial mediation
SER → UV → IB	0.38	Full mediation
SER → HV → IB		No effect

VAF, Variance accounted for; EU, Ease of use; VA, Visual appeal; SEC, Security; PT, Passing time; SER, Serendipity; UV, Utilitarian value; HV, Hedonic value; IB, Impulse buying.

With confirmation that the model lacked necessary conditions, the next step involved utilizing the truth table algorithm to predict the likelihood of variable combinations. Prior to sufficiency analysis, the data was preprocessed and frequency and raw consistency thresholds were established. Given this study’s substantial sample size, the frequency threshold was set to 3 while the raw consistency threshold was set to 0.8 to minimize the impact of the results’ antecedent conditions. The true table was organized based on frequency and consistency, describing the sufficient combinations for a high IB level.

The fsQCA results are listed in Table 8. The solution table encapsulates six combinations of constructs explaining why consumers exhibit high IB levels. The solution consistency was 0.81 above 0.8 and the solution coverage was 0.52 over 0.5, indicating the configurated solutions sufficiently explain the consumer behaviors associated with high IB in this study (Table 9).

**Table 8:** Analysis of necessary conditions of IB.

Conditions	Consistency	Coverage
EU (~EU)	0.63 (0.61)	0.71 (0.67)
VA (~VA)	0.66 (0.59)	0.69 (0.70)
SER (~SER)	0.65 (0.60)	0.69 (0.70)
SEC (~SEC)	0.65 (0.59)	0.70 (0.68)
PT (~PT)	0.66 (0.59)	0.69 (0.68)
UV (~UV)	0.61 (0.64)	0.69 (0.69)
HV (~HV)	0.63 (0.62)	0.68 (0.69)

EU, Ease of use; VA, Visual appeal; SEC, Security; PT, Passing time; SER, Serendipity; UV, Utilitarian value; HV, Hedonic value; IB, Impulse buying; ~, negation.

**Table 9:** Sufficient recipes to predict high IB.

Configuration	Solution					
	1	2	3	4	5	6
EU		●	•	●	●	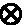
VA	●		●	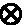	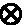	●
SER	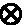	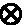	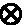	•	•	•
SEC		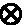	•		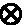	●
PT	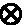	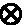	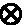	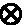	•	•
UV	•	●		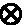	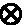	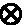
HV	●	●	●	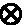	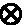	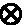
Raw coverage	0.287	0.215	0.229	0.177	0.203	0.248
Unique coverage	0.03	0.017	0.003	0.015	0.029	0.043
Consistency	0.862	0.833	0.892	0.901	0.901	0.901
Solution coverage	0.52					
Solution consistency	0.81					

EU, Ease of use; VA, Visual appeal; SEC, Security; PT, Passing time; SER, Serendipity; UV, Utilitarian value; HV, Hedonic value; IB, Impulse buying; •, Peripheral causal condition (present); ●, Core causal condition (present); 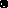, Peripheral causal condition (absent); 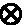, Core causal condition (absent); Blank, Do not care.

Solution 1 demonstrated that high VA, high hedonic value, and utilitarian value can induce high IB, aligning with the previous hypothesis. Solution 2 indicated that high EU, high utilitarian value, and high hedonic value can lead to high IB, implying that these three factors dominated user behaviors while other factors could be neglected. The results of Solution 1 and Solution 2 validates H6 and H7, where hedonic value and utilitarian value are influence to IB while EU and AV serve as important elements. Solution 3 indicated that high VA, high hedonic value, EU, and SEC can increase high IB. Solution 4 indicated that high EU and SER can cause high IB while other factors, especially hedonic value, could be disregarded. In this case, EU was the only core condition and was likely to dominate the other factors; the SCA’s availability appears to be an important factor influencing consumers’ IB. Solution 5 indicated that high EU, SER, and PT were the conditions leading to high IB. In such circumstances, consumers appear to engage in IB regardless of the level of utilitarian value and hedonic value. Solution 6 demonstrated that high VA, high SEC, SER, and PT can lead to high IB, consistent with the results of the PLS-SEM model. Solution 4, solution 5 and solution 6 altogether indicate that technical cues and situational cues are crucial factors in IB.

## 6 Discussion

### 6.1 General discussion

In this study, we adopted both symmetric and asymmetric analyses to investigate consumers experiencing boredom potentially engaging in IB on SCAs. Drawing from four theoretical perspectives – the S-O-R paradigm, social commerce, boredom theory, and IB theory – our research model extends the online IB model proposed by Parboteeah et al. (2009). Technical cues and situational cues were identified as crucial drivers of perceived values, which influence IB.

The results of this study suggest that EU, VA, and SEC are three key determinants of utilitarian value and hedonic value, supporting H1a-H3a and H1b-H3b. Furthermore, these values themselves were found to significantly influence IB, supporting H6-H7. When bored users browse SCAs, the technical advantages of SCAs (e.g., simple and clear operating systems, image-sharing features, a secure environment) impact them as consumers, triggering cognitive reactions (utilitarian) and affective reactions (hedonic) that stimulate IB.

Additionally, the results reveal a connection between PT and SER with utilitarian value (supporting H4a-H5a), while only PT significantly influenced hedonic value (supporting H5b). This suggests that PT can encourage consumers’ value perceptions, thereby driving IB. PT captures consumers’ attention and allows them to focus on product-related information, potentially mitigating their boredom as they concentrate on the SCA. Surprisingly, SER positively affected utilitarian value and hedonic value perceptions, though the impact on utilitarian value was significantly stronger. This implies that during impulsive consumption, consumer behavior not only relies on emotional awareness but also involves logical decision-making and cognitive deliberation (Hu et al., 2019).

When in a state of boredom, it is possible that utilitarian attributes are likely to improve consumers’ mood to the point of stimulating IB. This finding diverges from a previous study on SER in online IB in social commerce, which suggested a positive impact on IB (Bao and Yang, 2022). It appears that when consumers are bored (i.e., in a low emotional state), SER may not effectively stimulate their perceptions of hedonic value leading to IB. Furthermore, the mediation test results indicated that the effect of SER is fully mediated by utilitarian value. This implies that SER may not directly enhance IB unless the information about products reaches a certain level of users’ utilitarian needs. In essence, SER must be converted into utilitarian value to induce IB.

To the best of our knowledge, this is the first study to examine the influence of IB among bored consumers using the fsQCA method. Previous studies have been limited to the PLS-SEM method in exploring similar phenomena (Xia et al., 2024). Serving as a complement to the PLS-SEM approach, fsQCA revealed the occurrence of high IB levels through three sets of elements: technical cues, situational cues, and shopping values. Six configurations aligned with symmetric analytic findings, explaining the same results through different pathways, each with unique core conditions. For example, consumers only engage in IB if they perceive values in shopping, through the attraction to other attributes can still stimulate IB without generating perceptions of shopping values according to fsQCA.

### 6.2 Theoretical implications

This study significantly contributes to the existing study on IB in social commerce, particularly within the context of social commerce. The findings may offer valuable insights regarding consumer behavior during states of emotional distress over an online IB model. Previous research predominantly focused on IB driven by positive emotions (Yi and Jai, 2020), neglecting exploration of how consumers, when bored, develop the desire to consume. By adopting a conceptual model within the S-O-R framework, we bridge this gap and offers a quantitative perspective on the causes of impulsive consumption in moments of boredom.

Building on qualitative research by Sundström et al. (2019), who explored impulsive consumption in states of boredom based on in-depth qualitative interviews in the fashion industry, this study offers a quantitative investigation grounded in the S-O-R framework. It highlights the external technical factors of SCAs, emphasizing the importance of an environment conducive to impulse shopping. A positive shopping environment enables consumers to focus on the content of the SCA, creating a psychological sense of time passing quickly, thereby altering the bored consumer’s mindset and potentially leading to IB.

This study also classified the users’ perceived value in online shopping into utilitarian and hedonic categories. The results suggest that perceived values play a pivotal role in influencing IB. The study advances the understanding of how SCA users’ perceived value shapes their tendencies toward IB. When browsing in a state of boredom, utilitarian-oriented consumers prioritize products that satisfy certain needs, leading to IB. Conversely, hedonistic consumers seek pleasure culminating in IB. The direct impact of perceived values on IB underscores indicates that consumers aim to alleviate boredom at an everyday level rather than under extraordinary circumstances. These findings, centered on utilitarian and hedonic value, enrich the theoretical framework of consumer behavior and IB in the context of social commerce.

Finally, this study contributes to the understanding on consumer behavior during psychological states of boredom. While previous research has shown that visual stimulation and smooth user experiences on SCAs can stimulate IB (Zheng et al., 2019), few studies have integrated both psychological and technical factors into their research. Grounded in the S-O-R framework and featuring a clear definition of impulsive consumption, this study’s findings align with the proposed hypotheses. IB emerges as a coping mechanism for users attempting to escape boredom and monotony, offering an opportunity to “break free” from an “unpleasant reality.” When consumers feel bored, they may turn to browsing on SCAs as a means of passing time. The stimuli encountered during this casual browsing can stimulate IB. A previous study similarly demonstrated that college students tend to buy fashion items when experiencing boredom (Studak and Workman, 2004).

### 6.3 Practical implications

The results may provide valuable insights for SCA operators to enhance their systems and managerial strategies. The identified relationships between EU in SCAs and the utilitarian and hedonic value perceived by users underscore the importance of user fluency in SCA interactions. To capitalize on this, administrators can focus on optimizing tools that empower consumers to easily tag content of interest. Additionally, refining the system’s push algorithm can further personalize content recommendations, narrowing down items that align with users’ preferences.

Given the positive impact of VA on utilitarian and hedonic value, managers should also prioritize platform esthetics. Page layout, typography, and visual elements (e.g., color matching) should be strategically employed to highlight content and facilitate user navigation. Creative transformations in marketing content, leveraging visual neurostimulation, can capture users’ attention and foster impulsive consumption through repeated exposure.

Attention to the SEC is also crucial. Operators are urged to promptly address SEC risks. Implementing timely feedback mechanisms and robust privacy protection methods can safeguard users’ personal information, significantly influencing both utilitarian and hedonic value. Strengthening both types of perceived values enhances the potential for IB.

Recognizing the differences between offline and online consumers, particularly in their motivations to escape boredom (Sundström et al., 2019), offers an opportunity for SCA operators as well. Incorporating gamification, adventure-like programs, and rewards into the browsing process can engage users, providing effective stimuli that can encourage IB. Understanding the specific reasons behind consumers’ states of boredom may enable operators to tailor strategies for encouraging excitement and other positive emotions, ultimately driving the urge to buy products impulsively.

Creating an informative and useful platform is essential, as the impact of SER on utilitarian value emphasizes the importance of substance over mere interest. Emphasizing unique product features in ads and facilitating user-friendly search features through tags or exclusive multimedia content can attract consumers’ interest. Implementing a robust filtering system empowers users to easily sift through information, encouraging IB by highlighting high-quality, relevant content.

### 6.4 Limitation and future directions

While this research contributes to the understanding of IB, certain limitations warrant consideration. First, this study predominantly included respondents aged 20 to 30, potentially limiting the generalizability of its findings. Future investigations could explore IB similarities and variances across more diverse age groups. Second, the focus on understanding impulse purchases in the context of boredom may not have fully captured the multifaceted nature of social commerce, especially with emerging trends like live-streaming commerce. Subsequent research could delve into the dynamics of IB during live-streaming sessions. Third, this study relied on cross-sectional data collected through questionnaires, posing limitations on establishing causality and temporal dynamics. Future studies may adopt a longitudinal approach to overcome these constraints, enabling a more nuanced understanding of how IB unfolds over time and its applicability to diverse consumer groups. Addressing these considerations in future research can further enrich the understanding of IB within the evolving landscape of social commerce.

## 7 Conclusion

In this study, we investigated whether bored consumers engage in IB on SCAs through both qualitative and quantitative approaches using PLS-SEM and fsQCA. We explored the effects of technical and situational cues on IB, as well as the mediating role of consumers’ perceived shopping values. PLS-SEM revealed linear relationships between indicators from a quantitative perspective, while fsQCA uncovered the causal relationships between variables from a qualitative macro perspective, explaining high levels of IB in certain consumers by combining variables in various configurations. FsQCA also helped us test the feasibility of the results from symmetric analysis. The combination of these two methods facilitated an objective and accurate assessment of the proposed hypotheses. Our findings may provide valuable insights for SCA operators and brand managers, and can point toward innovative strategies to capture users’ sensations of boredom and fragmented time to stimulate IB.

## Data Availability

The original contributions presented in the study are included in the article/Supplementary material, further inquiries can be directed to the corresponding author.

## Footnotes

^1^www.wjx.cn
