# Supplementary material for: How technical and situational cues affect impulse buying behavior in social commerce? Evidence from bored consumers
Source: Front Psychol. 2024 Oct 2;15:1405189. doi: 10.3389/fpsyg.2024.1405189 (PMC11481336; doi:10.3389/fpsyg.2024.1405189)
Supplement: Supplementary file 1 [file Table_1.DOCX]

Supplementary Material

# Supplementary Tables

## Supplementary Figures

**Appendix A**

Appendix A Measuring items for research model

| Ease of use | |
| --- | --- |
| EU1 | Xiaohongshu is easy to use. |
| EU2 | My interaction with Xiaohongshu is clear and understandable. |
| EU3 | Learning to use Xiaohongshu is easy. |
| EU4 | It is easy to get Xiaohongshu to do what I want it to do. |
| Visual appeal | |
| VA1 | Xiaohongshu is visually pleasing. |
| VA2 | Xiaohongshu is visually pleasing design. |
| VA3 | Xiaohongshu is visually appealing. |
| Security | |
| SEC1 | Xiaohongshu is truthworth. |
| SEC2 | Xiaohongshu keeps payment enviornments security. |
| SEC3 | The products information on Xiaohongshu is credible. |
| SEC4 | I can shop on Xiaohongshu with confidence. |
| Passing time | |
| PT1 | I find a lot of exciting things to do on Xiaohongshu. |
| PT2 | I am only on Xiaohongshu when I have nothing better to do. |
| PT3 | When I am on Xiaohongshu I feel as if time is going by faster than usual. |
| PT4 | I am easily attracted on Xiaohongshu. |
| Serendipity | |
| SER1 | I obtained unexpected insights when doing shopping on Xiaohongshu. |
| SER2 | I unexpectedly discovered by chance what I want to buy before when doing shopping on Xiaohongshu. |
| SER3 | I found things that surprised me when doing shopping on Xiaohongshu. |
| SER4 | I was able to see the ordinary in new way when doing shopping on Xiaohongshu. |
| Utilitarian value | |
| UV1 | Using Xiaohongshu can improve my shopping performance (e.g., save shopping time or buying cost) in searching and buying products. |
| UV2 | Using Xiaohongshu can improve my shopping productivity (e.g., get good deals and products) in searching and buying products |
| UV3 | Using Xiaohongshu can improve my shopping efficiency in searching and buying products. |
| UV4 | Using Xiaohongshu enables me to better accomplish searching and buying products. |
| Hedonic value | |
| HV1 | I had fun using Xiaohongshu. |
| HV2 | I found my visit to Xiaohongshu interesting. |
| Urge to buy impulsively | |
| UBI1 | As I browse on Xiaohongshu, I had the urge to buy items other than or in addition to my specific shopping goal. |
| UBI2 | Browsing on Xiaohongshu, I had a desire to buy items that did not pertain to my specific shopping goal. |
| UBI3 | While browsing on Xiaohongshu, I had the inclination to purchase items outside my specific shopping goal. |

**Appendix B**

Appendix B Common method bias

|  | R1 | R1^2^ | R2 | R2^2^ |
| --- | --- | --- | --- | --- |
| EU1 | 0.823 | 0.677 | 0.067 | 0.004 |
| EU2 | 0.848 | 0.719 | -0.015 | 0.000 |
| EU3 | 0.808 | 0.653 | -0.018 | 0.000 |
| EU4 | 0.833 | 0.694 | 0.034 | 0.001 |
| VA1 | 0.835 | 0.697 | 0.309 | 0.095 |
| VA2 | 0.847 | 0.717 | 0.315 | 0.099 |
| VA3 | 0.832 | 0.692 | 0.014 | 0.000 |
| SEC1 | 0.830 | 0.689 | -0.026 | 0.001 |
| SEC2 | 0.815 | 0.664 | -0.001 | 0.000 |
| SEC3 | 0.802 | 0.643 | -0.024 | 0.001 |
| SEC4 | 0.821 | 0.674 | 0.006 | 0.000 |
| PT1 | 0.832 | 0.692 | 0.006 | 0.000 |
| PT2 | 0.790 | 0.624 | -0.039 | 0.002 |
| PT3 | 0.808 | 0.653 | 0.007 | 0.000 |
| PT4 | 0.802 | 0.643 | 0.044 | 0.002 |
| SER1 | 0.822 | 0.676 | 0.003 | 0.000 |
| SER2 | 0.834 | 0.696 | -0.065 | 0.004 |
| SER3 | 0.798 | 0.637 | -0.007 | 0.000 |
| UV1 | 0.813 | 0.661 | 0.019 | 0.000 |
| UV2 | 0.835 | 0.697 | -0.014 | 0.000 |
| UV3 | 0.839 | 0.704 | 0.304 | 0.092 |
| UV4 | 0.840 | 0.706 | 0.357 | 0.127 |
| HV1 | 0.886 | 0.785 | -0.026 | 0.001 |
| HV2 | 0.885 | 0.783 | 0.026 | 0.001 |
| IB1 | 0.804 | 0.646 | -0.011 | 0.000 |
| IB2 | 0.827 | 0.684 | -0.009 | 0.000 |
| IB3 | 0.799 | 0.638 | -0.008 | 0.000 |
| IB4 | 0.812 | 0.659 | 0.033 | 0.001 |
| AVG |  | 0.682 |  | 0.015 |
